# When Is the Negative Effect of Abusive Supervision on Task Performance Mitigated? An Empirical Study of Public Service Officers in Korea

**DOI:** 10.3390/ijerph17124244

**Published:** 2020-06-14

**Authors:** Heetae Park, Wonseok Choi, Seung-Wan Kang

**Affiliations:** 1 College of Business Administration, Dong-A University, Busan 49237, Korea; mtree@dau.ac.kr; 2 College of Business Administration, University of Detroit Mercy, Detroit, MI 48221, USA; 3 College of Business, Gachon University, Seongnam 13120, Korea

**Keywords:** abusive supervision, task performance, coworker support, self-efficacy, public service officer, military

## Abstract

Supervisory leadership has occupied an important place in management literature in identifying the supervisory behaviors that are associated with positive outcomes. However, researchers also have turned their attention to the dark side of supervisory behavior, such as abusive supervision. This study investigates the role of coworker support and self-efficacy in the relationship between abusive supervision and the subordinate’s task performance. Data are collected from 192 supervisor–subordinate pairs in the South Korean Army. As hypothesized, when subordinates receive higher levels of coworker support or have higher self-efficacy, abusive supervision is less negatively related to task performance. The implications of the study and directions for future research are discussed.

## 1. Introduction

Leaders do not always behave in a beneficial and constructive manner; they often behave in a negative and ineffectual manner. Negative leadership behaviors are harmful enough to cancel any beneficial effectiveness of the favorable actions of leaders and undermine the influence on the positive behaviors of the subordinate [1,2]. Many scholars have investigated abusive supervision as destructive leadership [3,4,5,6,7,8]. Abusive supervision, for example, harms the subordinates’ organizational social consciousness and creativity, which are critical for the success of organizations [8,9]. Hargreaves and Fink [10] argued that unfair infringement of human resources, such as abusive supervision, is a serious threat to the organization’s heathy environment.

Nevertheless, the focus of most leadership studies has remained on effective and exemplary behavior, and negative leader behaviors need more attention in academia [11,12]. Abusive supervision has been reported to have a negative effect on the task performance of subordinates [13,14,15,16]. However, the relationship between abusive supervision and task performance is still being explored. Glomb and Hulin [17], for instance, found that a supervisor’s anger led subordinates to perform better. Lee, Yun, and Srivastava [18] demonstrated the existence of a curvilinear (inverted U-shaped) relationship between a supervisor’s abusive supervision and an employee’s creative performance. These studies call for more attention by researchers to the relationship between abusive supervision and performance. Particularly, research into the moderating factors is necessary to attenuate the negative impact of abusive supervision on subordinate work efficiency.

Considering this, we focus on conditional factors that may moderate the relationship between abusive supervision and performance, such as coworker support and self-efficacy. Self-efficacy, which is defined as an individual’s faith in his or her own capability to carry out a specific task [19], plays a critical role in shaping one’s response to a supervisor’s abusive behaviors. When people have faith in their own ability, they may resist negative treatment by a supervisor [20]. Moreover, it has been suggested that emotional support from colleagues may help subordinates overcome stress and difficulty in an organization [21]. Given that subordinates interact with each other as much as or more often than they do with their supervisors [22], colleagues can be an important source of social support, and this support will play an important role in relieving the negative effect of abusive supervision. Thus, we examine the moderating roles of coworker support and self-efficacy to mitigate the negative impact of abusive supervision on the task performance of subordinates.

**Figure 1:**
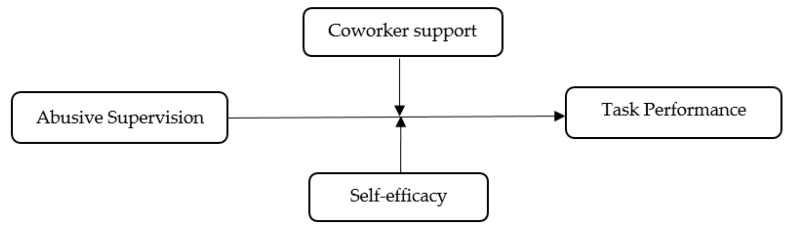
Research model.

Our research model is presented in Figure 1.

## 2. Theoretical Background and Hypotheses

### 2.1. Abusive Supervision and Task Performance

Abusive supervision is the “subordinates’ perceptions of the extent to which supervisors engage in the sustained display of hostile verbal and nonverbal behaviors, excluding physical contact” [23]. Studies have not always found a consistent relationship between the abusive behaviors of supervisors and the subordinates’ task performance. Some studies have shown that abusive supervision can result in either positive or negative task performance [13,17]. One possible factor causing the mixed results is the resources that subordinates use to deal with abusive supervision.

Conservation of resources (COR) theory suggests that individuals protect their current personal resources and get new resources to achieve their goals. However, when these resources are lost or threatened, individuals must invest resources to protect against resource loss, recover from losses, and gain resources [24,25]. Hobfall, Halbesleben, Neveu, and Westman [26] found that those with greater resources are less vulnerable to resource loss and more capable of resource gain. Conversely, individuals and organizations that lack resources are more vulnerable to resource loss and less capable of resource gain. Under the circumstances of dealing with abusive supervision, those subordinates with greater resources are less vulnerable to resource loss and more capable of resource gain. They tend to perform better. Conversely, if those subordinates lacking resources are more vulnerable to resource loss and less capable of resource gain, it may have a negative effect on task performance. Considering this, we examine the potential influence of coworker support and self-efficacy on the subordinate’s perception of abusive supervision, which may determine the consequence of abusive supervision.

### 2.2. Moderating Effect of Coworker Support and Self-Efficacy

Coworker support is a type of social support that includes various forms of assistance and emotional consolation from colleagues [27]. House [28] defines social support as the perception and actuality that one is cared for, has assistance available from other people, and that one is part of a supportive social network. Social support has several dimensions such as instrumental support, informational support, and emotional support. However, many researchers mainly have focused on the providers’ instrumental and emotional support [29,30,31,32,33]. Instrumental support is to provide tangible goods, services, or tangible information to another during a time of stress, while emotional support is the provision of caring, empathy, and trust.

When employees collectively experience psychological pressure from supervisors, their coworkers can provide emotional support which consoles them, and coworker support can reduce the pressure and decrease its negative effect [34,35]. Additionally, coworkers in a similar or related position can provide instrumental support such as practical information and advice about the task and career. Despite these benefits of coworker support [22], limited attention has been paid to the notion that coworker support can play an important role in complementing deficient support from abusive supervisors.

Studies have shown that coworker support increases affection and commitment [34,35,36,37] and that coworker cooperation, information provision, and emotional support relieve the negative effect of stress from the work environment [2,37]. Likewise, coworker support may offset the loss of psychological resources due to abusive supervision.

Given this, a subordinate who receives enough support from colleagues will have more resources to deal with abusive supervision effectively. Conversely, when coworker support is low, and a supervisor’s abusive supervision is high, subordinates will receive insufficient support from colleagues to compensate for the aggressive behaviors of supervisors. Under such conditions, subordinates will experience the severe loss of psychological resources, which results in low performance. Coworker support is a specific environmental resource to reduce the negative effect of abusive supervision. Hobfoll, Freedy, and Geller [38] suggest that social support may be a central building block of health and well-being because, together with personal resources, it is related to an overall sense of identity, for instance. Halbesleben [39] found the resource effects of social support to be a moderator. Wu and Hu [40] found that the moderating effect of perceived coworker support showed the existence of a stronger relationship between abusive supervision and emotional exhaustion when coworker support was high. Thus, we expect the following:

**Hypothesis** **1.** *Coworker support will moderate the relationship between abusive supervision and the subordinate’s task performance, such that when coworker support is high, abusive supervision will not be negatively related to a subordinate’s task performance, but when coworker support is low, abusive supervision will be negatively related to a subordinate’s task performance.*

Individual differences have been known to influence the interpretation of and reaction to supervisor behaviors [41,42,43]. Among those individual differences, we focus on self-efficacy. Self-efficacy is defined as “an individual faith in one’s own capability to carry out a special task” [19]. Wood and Bandura [44] suggested that self-efficacy influences one’s perception of the current condition and the selection, implementation, and persistence of action. Likewise, many scholars have asserted that self-efficacy incites the desire to accomplish, reduces stress, lowers negative emotions such as depression, and improves individual accomplishment and well-being [45,46,47,48,49]. Self-efficacy is a specific cognitive resource that could decrease the effects of abusive supervision. Kluemper et al., [50] demonstrate the moderating role of cognitive ability between abusive supervision and the employee’s deviant reactions. Considering this, those with high self-efficacy are less vulnerable to resource loss and more capable of resource gain for task performance. Conversely, those with low self-efficacy are more vulnerable to resource loss and less capable of resource gain for task performance. Thus, we expect the following:

**Hypothesis** **2.** *The subordinate’s self-efficacy will moderate the relationship between abusive supervision and the subordinate’s task performance, such that when self-efficacy is high, abusive supervision will not be negatively related to task performance, but when self-efficacy is low, abusive supervision will be negatively related to the subordinate’s task performance.*

## 3. Methods

### 3.1. Participants and Data Collection

We tested our hypotheses on a sample of military officers in South Korea. The questionnaires were distributed to 290 non-commissioned officers who were squad commanders (i.e., subordinates) and to their direct superiors who were platoon leaders (i.e., supervisors). A platoon leader evaluated only one squad commander; therefore, none of the subordinates were nested within the same supervisor workgroup. This study was reviewed and approved by the research review board of the National Defense University prior to the data collection, and it followed the guidelines of the International Declaration of Helsinki. During the survey phase, the purpose and procedure of the study were explained to all survey subjects. We informed them that their participation in the survey should be entirely voluntary, and they were free to quit at any time if they wished. Finally, informed consent was obtained from the participants. Regarding confidentiality purposes, each group, one subordinate and one supervisor, answered their questionnaires at a different time and in a different place. Among them, pairs returned their questionnaires, but after excluding the respondents who had skipped more than 50% of the questions, we arrived at 192 subordinate–supervisor pairs.

Ninety eight percent of all subordinates and 99% of all supervisors were males. The average age of the supervisors was 24.5 years and that of the subordinates was 23.2 years. The average job tenure of the supervisors was seven months. All supervisors were officers, and 65.8% of them were second lieutenants. Eighty seven percent (173) of the subordinates were noncommissioned staff sergeants.

### 3.2. Measures

All questions used the five-point Likert-type scale (1 = strongly disagree to 5 = strongly agree). To minimize the common-method variance, all questions related to independent variables (i.e., abusive supervision, coworker support, and self-efficacy) were answered by the subordinates, and the dependent variables (i.e., subordinate’s task performance) were measured by their immediate supervisor’s response.

#### 3.2.1. Abusive Behaviors of Supervisors

Abusive behaviors of supervisors were measured using Tepper’s 15 items [23]. This scale measures the degree of perception by subordinates of the specific behaviors of supervisors described in the questions. A sample item is “My supervisor makes a fool out of me”. (α = 0.98).

#### 3.2.2. Task Performance of Subordinate

A subordinate’s task performance was evaluated by their immediate supervisors using the seven items of Williams and Anderson [51]. Sample items include “Subordinate completes the given task properly”, “Subordinate fulfills the assigned task and responsibility”, and “Subordinate carries out the task that is promising to himself”. (α = 0.89).

#### 3.2.3. Coworker Support

Coworker support was measured using Baruch–Feldman et al.’s seven items [27]. A sample item is “My colleagues have shown their concern about me”. (α = 0.92).

#### 3.2.4. Self-Efficacy

A subordinate’s self-efficacy was measured using the eight items of Chen, Gully, and Eden [52]. A sample item is “I can accomplish most of my setup goals”. (α = 0.95).

### 3.3. Analytical Approach

We proceeded with the normal moderated regression tests. Specifically, the hypothesized models were tested through ordinary least squared (OLS) regression. To rule out spurious relationships, three demographic variables (i.e., age, rank, and tenure with supervisor) were entered as additional predictors in a regression analysis. Concerning the test of moderation in Hypotheses 1 and 2, we followed the procedure outlined by Aiken and West [53]. Prior to calculating the multiplicative interaction terms, we centered the independent variables at the mean [53]. To reduce the number of terms included in each regression model, thereby increasing the likelihood of detecting significant effects, we tested the hypotheses involving moderating effects in separate regression models. First, we entered the control variables followed by an independent variable and a moderator. Then, we entered the product between an independent variable and a moderator and checked whether the product term was significant.

## 4. Results

Table 1 shows the means, standard deviations, and correlations among variables. Abusive supervision is negatively correlated to task performance of subordinates (*r* = −0.34, *p* < 0.01), coworker support (*r* = −0.47, *p* < 0.01), and self-efficacy, respectively (*r* = −0.60, *p* < 0.01). Task performance of subordinates, on the other hand, is positively correlated to coworker support and self-efficacy, respectively (*r* = 0.49, *p* < 0.01; *r* = 0.38, *p* < 0.01).

**Figure 2:**
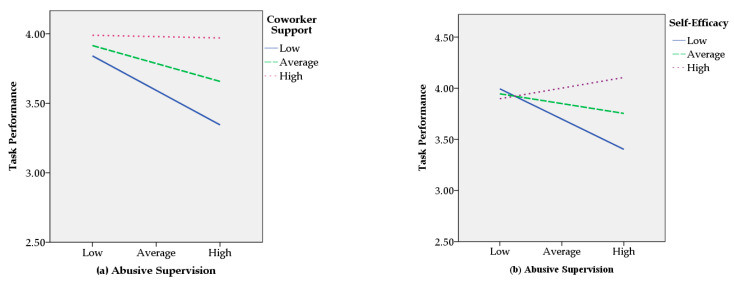
(**a**) Interaction Plot of Abusive Supervision and Coworker Support; (**b**) Interaction Plot of Abusive Supervision and Self-Efficacy.

The results of the regression analyses are shown in Table 2. Hypothesis 1 proposes that when coworker support is low, abusive supervision will be negatively related to the subordinate’s task performance, but when coworker support is high, abusive supervision will not be negatively related to the subordinate’s task performance. Seen in Model 2 of Table 2, the coefficient of this moderation effect is significant (*β* = 0.15, *p* < 0.01). Figure 2a depicts the interaction plot [45]. A simple slope test indicates that when coworker support is low, abusive supervision is negatively related to the subordinate’s task performance (−1 *SD*; *b* = −0.24, t = −2.96, *p* < 0.01), but when coworker support is high it is not significantly related to task performance (+1 *SD*; *b* = −0.01, *t* = 0.10, *p* = 0.92). This pattern of results provides support for Hypothesis 1.

Hypothesis 2 predicted that the subordinate’s self-efficacy will moderate the relationship between abusive supervision and the subordinate’s task performance, such that when self-efficacy is low, abusive supervision will be negatively related to task performance, but when self-efficacy is high, abusive supervision will not be negatively related to the subordinate’s task performance. Model 3 of Table 2 confirms that the coefficient for this moderation is significant (*β* = −0.25, *p* < 0.01). Seen in Figure 2b, abusive supervision is negatively related to task performance for those with low self-efficacy (−1 *SD*; *b* = −0.29, *t* = −5.24, *p* < 0.01), but it is not significantly related to task performance for those with high self-efficacy (+1 *SD*; *b* = 0.10, *t* = 1.76, *p* = 0.08). Thus, Hypothesis 2 is supported.

## 5. Discussion

During this study, we further explored the relationship between abusive supervision and task performance by examining the moderating effect of coworker support and self-efficacy. Our results show that the influence of abusive supervision on subordinate performance varies with coworker support and self-efficacy. Consistent with our expectation, we found that when coworker support or self-efficacy is low, abusive supervision is more negatively related to task performance.

### 5.1. Theoretical and Practical Implications

We have verified the moderating effect of perception of coworker support and self-efficacy of subordinates in relation to abusive supervision and the subordinate’s task performance. First, our findings imply that abusive supervision will have more harmful effects on subordinate performance if subordinates have fewer resources, such as coworker support and self-efficacy. Therefore, organizations should consider the supportive climate of the work unit and the role of the subordinate’s self-efficacy. Organizations should develop training in leadership programs and help supervisors develop suitable managerial behavior to be aware of their subordinates’ relationships with colleagues and their cognitive ability.

Second, this study shows the importance of self-efficacy, which involves determination and perseverance to overcome obstacles that would interfere with achieving goals. Studies have shown that when a supervisor engages in abusive behavior, the subordinate’s task performance declines. However, we found that the subordinates with high self-efficacy performed better than subordinates with low self-efficacy, and their performance did not decline even under abusive supervision. This suggests the possibility that self-efficacy provides a resource that a subordinate can use to endure the negative impact of abusive supervision. Thus, a subordinate’s strong self-efficacy can be seen as not only a source of motivation to make further efforts, but also a resource that helps in coping with a supervisor’s hostile verbal and nonverbal behaviors.

### 5.2. Limitations and Future Research

First, this study is based on a cross-sectional design and has a limitation in its cause-and-effect relationship. Andersson and Pearson [54] suggested negative reciprocity principles, which means that supervisors and subordinates can be mutually affected by negative behavior [55]. Thus, this study cannot completely exclude the possibility of a reverse causal relationship between abusive supervision and task performance. Future research based on longitudinal design is required. Second, the sample of this study is from the South Korean Army, which has a very strong hierarchical relationship between primary officers and squad commanders. To generalize our findings, it is necessary to verify if the same phenomenon occurs in a general company or a less hierarchical organization as well. Third, it will be interesting to examine the deviant behavior [56] of subordinates with two moderators: coworker support and self-efficacy, found in this study. Abusive supervisory behaviors increase deviant behaviors of subordinates, and it will be interesting to examine what roles coworker support and self-efficacy play in the relationship between abusive supervision and deviant behavior. Finally, it is necessary to investigate the influence of leadership in opposite-sex leader–subordinate dyads [57]. Vecchio and Brazil [58] found that same-sex leader–subordinate pairings had more positive working relationships than different-sex pairings in the military. Future research is required to study the effects of abusive supervision on task performance, through a survey of pairs of men and women differently.

## 6. Conclusions

Our study provides important implications by verifying two moderators: coworker support and self-efficacy, which can attenuate the negative influence of abusive supervision on the subordinate’s task performance.

## Figures and Tables

**Table 1:** Bivariate Correlations and Reliabilities.

Variable	M	SD	1	2	3	4	5	6	7
1. Age	22.02	1.47							
2. Rank	2.81	0.91	0.40 **						
3. Tenure with supervisor	2.03	0.81	0.32 **	0.54 **					
4. Abusive supervision	2.02	1.03	0.18 *	0.44 **	0.32 **	(0.98)			
5. Coworker support	3.90	0.79	−0.33 **	−0.18 *	−0.18 *	−0.47 **	(0.92)		
6. Self-efficacy	3.77	0.77	−0.23 **	−0.23 **	−0.25 **	−0.60 **	0.74 **	(0.95)	
7. Task performance	3.73	0.71	−0.31 **	−0.17 *	−0.14	−0.34 **	0.49 **	0.38 **	(0.89)

Note: *N* = 192, * *p* < 0.05, ** *p* < 0.01, Numbers in parentheses mean the reliability of each variable (Cronbach’s *α*); M = Mean, SD = Standard deviation.

**Table 2:** Result of Stepwise Ordinary Least Squared (OLS) Regression Analyses.

Variables	Task Performance of Subordinates		
	Model 1	Model 2	Model 3
Constant	7.05 ** (0.72)	5.12 ** (0.64)	5.23 ** (0.70)
**Stage 1: Control Variables**			
Age	−0.14 ** (0.04)	−0.07 * (0.03)	−0.07 (0.03)
Rank	0.06 (0.07)	0.03 (0.06)	0.06 (0.06)
Tenure with supervisor	0.02 (0.07)	0.03 (0.06)	0.03 (0.06)
**Stage 2: Independent Variable**			
Abusive supervision	−0.23 ** (0.05)	−0.12 ** (0.05)	−0.09 (0.06)
**Stage 3: Moderators**			
Coworker support		0.25 **(0.08)	
Self-efficacy			0.20 (0.08)
**Stage 4: 2-Way Interaction Terms**			
Abusive supervision X Coworker support		0.15 ** (0.05)	
Abusive supervision X Self-efficacy			0.25 ** (0.07)
*R* ^2^	0.19	0.31	0.28
*F* change		7.65 **	14.89 **
*R*^2^ change		0.03	0.06

Note: *N* = 192, * *p* < 0.05, ** *p* < 0.01; values are unstandardized coefficients.
